# Effect of enzymatic saccharification on edible seaweed (*Ulva* sp.) fermentation

**DOI:** 10.1007/s00253-026-13864-4

**Published:** 2026-05-28

**Authors:** João Reboleira, Susana F. J. Silva, Keshavan Niranjan, Young-Jung Wee, Mi-Kyung Park, Marco F. L. Lemos, Afroditi Chatzifragkou

**Affiliations:** 1 MARE - Marine and Environmental Sciences Centre, ARNET—Aquatic Research Network Associated Laboratory, ESTM, Polytechnic of Leiria, Peniche, 2520-641 Portugal; 2 Department of Food and Nutritional Sciences, University of Reading, Whiteknights Campus, Reading, RG6 6DZ UK; 3 Department of Food Science and Technology, Yeungnam University, Gyeongsan, 38541 Republic of Korea; 4 School of Food Science and Biotechnology, Food and Bio-Industry Research Institute, Kyungpook National University, Daegu, 41566 Republic of Korea

**Keywords:** *Cyberlindnera jadinii*, *Lactiplantibacillus plantarum*, Saccharification, Seaweed fermentation, *Ulva* sp.

## Abstract

**Abstract:**

Edible seaweeds are a promising substrate for microbial fermentations and the development of novel food products. However, the unique composition of their cell wall can limit microbial growth. To address this, the edible seaweed *Ulva* sp. was subjected to enzymatic saccharification using: (i) a crude extract of *Aspergillus oryzae* grown on *Ulva* sp.; (ii) a commercial enzymatic cocktail; and (iii) a combination of both. Saccharification with the latter substantially increased the release of fermentable sugars, with glucose (157.08 mg/g_substrate_) and galacturonic acid (153.83 mg/g_substrate_) as the dominant products, indicating disruption of key structural polysaccharides. Follow-up fermentations with *Lactiplantibacillus plantarum* and *Cyberlindnera jadinii* (GRAS microorganisms) exhibited a two-log growth increase compared to cultures in non-saccharified media. Saccharification also led to increased acidification and free amino nitrogen release by *L. plantarum*, and an increased nitrogen intake by *C. jadinii*, changes directly relevant to food fermentations. Despite these advances, the limited release of certain sugars suggests that some cell wall components remain resistant to hydrolysis. Overall, this work highlights enzymatic saccharification as a key enabling step for converting *Ulva* sp. into a viable and functional substrate for microbial fermentation and novel food development.

**Key points:**

• *A. oryzae extract combined with cellulolytic enzymes led to highest sugar yield*.

• *Saccharification improved growth, FAN release, and acidification by L. plantarum*.

• *Evidence of cellulose hydrolysis but not of the remaining cell wall polysaccharides*.

**Graphical abstract:**

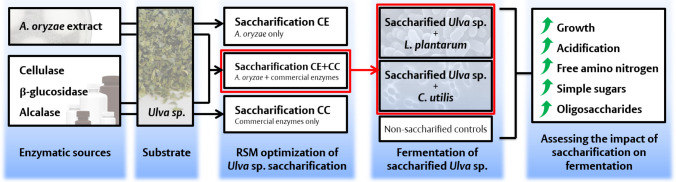

**Supplementary Information:**

The online version contains supplementary material available at 10.1007/s00253-026-13864-4.

## Introduction

Seaweeds are generally regarded as a poor substrate for most conventional, terrestrial food-fermenting cultures due to the difficulty in breaking down their cell wall constituents (Maneein et al. 2018). This limits the availability of assimilable sugars and can lead to nitrogen bottlenecks, since peptides and amino acids remain locked in the inaccessible cytoplasm, restricting microbial growth (Milledge and Harvey 2016). With regards to the edible seaweed *Ulva* sp., a significant part of the cell wall is made of ulvan, a sulfated polysaccharide which accounts for approximately 10–17% of total dry weight in various *Ulva* species (Kidgell et al. 2019). Ulvan is a polyanionic heteropolysaccharide with a backbone of repeated disaccharide units referred to as ulvanobiuronic acids type A_3s_ (β-D-glucuronic acid (1,4)-linked to α-L- rhamnose 3-sulfate), and type B_3s_ (α-L-iduronic acid (1,4)-linked to α-L-rhamnose 3-sulfate), as well as ulvanobioses type U_3s_ (β-D-xylose (1,4)-linked to α-L-rhamnose 3-sulfate) and type U_2′s,3 s_ (β-D-xylose 2-sulfate (1,4)-linked to α-L-rhamnose 3-sulfate), in order of frequency, and with types A and B being more common than types U (Kidgell et al. 2019; Wahlström et al. 2020; Li et al. 2023). Despite its thorough structural elucidation, the fermentability of ulvan by food-fermenting cultures is not particularly well investigated and understood (Gotteland et al. 2020; Li et al. 2023). Attempts to understand the potential prebiotic role of ulvan led researchers to conclude that glycosidic bonds and the unique sequence of sugars, as opposed to the sulfate or uronic acid content, were responsible for its poor fermentability (Bobin-Dubigeon et al. 1997a). Furthermore, cellulose, beta-(1,4)-xyloglucans and beta-(1,4)-glucuronans constitute the remainder of the cell wall in *Ulvales* (Wahlström et al. 2020; Li et al. 2023). Bobin-Dubigeon et al. (1997a, b) showed that these polysaccharides are highly resistant to bacterial degradation, likely due to the abundance and mesh-like layout of ulvan in the cell wall shielding the remaining glucans (Bobin-Dubigeon et al. 1997b).

Pretreatment strategies such as acid/alkaline hydrolysis, heat treatment, milling, ultrasonic disruption, and enzymatic digestions have been investigated as means to improve fermentation yields in a variety of seaweed species (Maneein et al. 2018; Podolean et al. 2022). Acid hydrolysis and heat treatments are a popular approach due to their simplicity and reliable impact on soluble sugar content (Maneein et al. 2018). Their use leads to the accumulation of furfural and hydroxymethylfurfural (HMF), known microbial inhibitors that are products of the dehydration of sugars (Giacon et al. 2022). This leads to a limit on how much these processes can benefit fermentation and has led to a growing reliance on enzymatic processes either as an alternative or used in conjunction (Brandão et al. 2025). Seaweed hydrolysis by cellulase has been reported to improve fermentability and production of lactic acid by *Lactobacillus acidophilus* and *Lactobacillus plantarum* by approximately 45% compared to the control (Lin et al. 2020). Authors Ramachandra and Hebbale (2020) also reported that cellulase pre-digestion is essential for bioethanol production from a variety of seaweeds, including *Ulva* sp. However, the impact of enzymatic saccharifications on seaweed food fermentations is less well reported and the limits of traditional food fermentations, when applied to seaweeds as a substrate, remain underreported.

This study addresses these gaps by evaluating how enzymatic saccharification influences the fermentability of *Ulva* sp. and the metabolic outcomes of its subsequent fermentation by *Lactiplantibacillus plantarum* and *Cyberlindnera jadinii*. Different sources of hydrolytic enzymes were employed, including a crude extract from *Aspergillus oryzae*, a commercial cellulolytic cocktail, and a combined mixture of the two. The use of these extracts in the saccharification of *Ulva* sp., as well as their effects on the growth of generally regarded as safe (GRAS) microorganisms, are novel highlights of this study. Insights on the use of *Ulva* sp. carbohydrates by these cultures were also provided, illuminating future steps for further improvements on similar processes for species of the *Ulva* genus.

## Materials and methods

### Microorganisms and inoculum preparation

*Cyberlindnera jadinii* DSM 2361 and *Aspergillus oryzae* DSM 1862 were purchased in freeze-dried form from Leibniz Institute DSMZ (Germany), while *L. plantarum* NCDO 1752 was *sourced* from the Department of Food and Nutritional Sciences culture collection at the University of Reading. Upon revival, *Cyberlindnera jadinii* and *Aspergillus oryzae* strains were grown on Potato Dextrose Agar (PDA) plates at 30 °C for 3 days and were kept at 4 °C until further use. *L. plantarum* cultures were grown in MRS agar plates at 37 °C for 24 h and kept at 4 °C until further use. For fermentation inoculum preparation, 48-h cultures of *C. jadinii* were collected and suspended in a 0.8% (w/v) NaCl, up to an OD_600_ of 0.1 (Abs). In the case of *A. oryzae* inoculum, 10 mL of 0.8% (w/v) NaCl with 0.1% (w/v) Tween-20 were added into 72-h agar plates and scraped with a sterile metal loop. The suspension was then collected and diluted to an OD_600_ of 0.1. Finally, in the case of *L. plantarum,* a full loop of 24 h colonies was suspended in 0.8% (w/v) NaCl to an OD_600_ of 0.1.

### Seaweed media preparation

Dried and desalinized flakes (<10 mm) of laminar *Ulva* species (*Ulva* sp.), purchased from Algaplus (Ílhavo, Portugal), were ground using a coffee grinder to an average particle size of approximately 1 mm. For each fermentation set, 2 g of ground flakes were hydrated using 40 mL of deionized water (1:20 solid-to-liquid ratio) in 100 mL Duran bottles. The pH of the resulting slurry was adjusted to 6.0 using 100 mM HCl and sterilized at 121 ºC for 15 min. Upon cooling, each bottle was inoculated with 0.8 mL of each culture’s suspension.

### Non-saccharified semi-solid *Ulva* sp. fermentations

Non-saccharified *Ulva* sp. slurries were fermented with *C. jadinii*, *A. oryzae,* and *L. plantarum*. Fermentations were conducted at 30 °C (*C. jadinii* and *A. oryzae*) and 37 °C (*L. plantarum*) at an agitation rate of 180 ± 5 rpm in an orbital shaker over a maximum period of 96 h. A set of sterilized, non-inoculated *Ulva* sp. slurries was also added on the orbital shaker and used as unfermented controls. Sampling took place at 0, 6, 12, 24, 48, 72, and 96 h after inoculation and for each sampling point three flasks were collected (triplicate independent measurements, *n* = 3). From each flask, 1 mL of slurry was aseptically recovered and used for viable colony counts via serial dilutions. The remaining volume was used for pH measurement and then centrifuged (11,600 × *g*) for 10 min. The supernatant was recovered and stored at −20 ºC until further analysis. Supplementary Figure S1 includes a flowchart of the fermentation methodology used in this study.

### Optimization of *Ulva* sp. enzymatic saccharification

The enzymatic saccharification of *Ulva* sp. was optimized using a response surface methodology (RSM). The influence of three factors: substrate load (SL), enzymatic load (EL), and saccharification time (t), on the concentration of reducing sugars (RS) and free amino nitrogen (FAN) was evaluated. A Box-Behnken design with three center points and a total of 15 runs was employed for each set of the optimization experiment. The setup of independent variables and their levels is presented in Table S1.

The supernatants of 48 h-fermented *Ulva* sp. cultures with *A. oryzae* were selected as crude extract with the highest saccharifying potential, were labelled as “crude extract” (CE), and used in Set 1. A cocktail of commercially acquired cellulolytic enzymes (CC) was employed in Set 2. These included cellulase (700 U/mL) (Celluclast 1.5 L), β-glucosidase (7 U/mg), and alcalase (0.75 U/mL), all procured from Novozyme, Bagsvaerd, Denmark. When combined with the reaction media, the applied enzymatic loads were 7, 70, and 133 U/g_substrate_ for cellulase, 0.7, 7, and 13.3 U/g_substrate_ for β-glucosidase, and a fixed 0.25 U/g_substrate_ for alcalase, following similar experiments by (Lee et al. 2012; Trivedi et al. 2013; Kim et al. 2023). Set 3 comprised the same enzymatic blend with the addition of *A. oryzae* crude extract (CE + CC), maintaining the same enzymatic loads as in previous sets. Table S2 (supplementary material) shows a complete list of all conditions tested, with the quantities of each individual component per run for the third set (CE + CC).

Enzymes were individually added to sterilized (121 ºC, 15 min) batches of *Ulva* sp. slurry in 0.1 M sodium citrate buffer at pH 6.0. Incubations took place in a shaking incubator at 50 °C and a constant agitation rate of 180 ± 5 rpm. Hydrolysis was stopped by quenching saccharified slurries in boiling water (100 °C) for 5 min; they were then quickly cooled down using an ice bath, centrifuged (11,600 × *g*), and the supernatants were stored at −20 °C until further analysis.

Optimized conditions were identified using the critical points representing maximum reducing sugar output. For factors where the theoretical maximum landed outside the design space, conditions were set near the edge of their verified maximums.

### Saccharified semi-solid *Ulva* sp. fermentations

Two additional batches of *Ulva* sp. slurry, with approximately 500 mL each of 0.1 M sodium citrate buffer, pH 6.0, were saccharified under optimized conditions. Unlike previous saccharifications, this batch was only heat-treated after the enzymatic incubation was completed to avoid processing the substrate twice. Once cooled down, the saccharified media was split into smaller vessels and inoculated with either *L. plantarum* or *C. jadinii*, in triplicate. The fermentation conditions and sampling routine were performed as described for the non-saccharified *Ulva* sp.

### Analytical methods

#### Viable colony counts in semi-solid fermentations

Microbial growth was monitored via plate counts. Specifically, one mL of slurry was serially diluted (10^5^-fold for *A. oryzae*, 10^6^-fold for *C. jadinii* or 10^8^-fold for *L. plantarum*) using sterile 0.8% (w/v) NaCl. *A. oryzae*-fermented slurries were blended in sterile stainless steel blender cups before sampling to break down the mycelium clumps formed during shake-flask fermentation. Selected dilutions, contingent on fermentation duration, were transferred and spread onto PDA plates for yeast and mould cultures or incorporated into MRS agar in the case of *L. plantarum*. Plates were incubated for 48 h either at 30 °C (*C. jadinii* and *A. oryzae*) or 37 °C (*L. plantarum*), after which colony counts were enumerated. Results were expressed in log colony-forming units per mL of slurry (log CFU/mL). pH was measured in tandem with the sampling of the fermented slurry using the leftover volume and a benchtop potentiometer.

#### Protein and free amino nitrogen (FAN) analysis

Total protein in unprocessed, dried *Ulva* sp. flakes was determined using the Kjeldahl method, with a nitrogen conversion factor of 5.00 (Angell et al. 2016). Data was expressed as percent of crude protein per kg of dry seaweed, with analysis being performed in triplicates. FAN was determined using the colorimetric ninhydrin method described by Lie (1973), with glycine (0.25 to 2.5 mg/L) being used as standard and results presented as mg of glycine equivalents per g of dry substrate (mg_Gly(eq)_/g_substrate_). The Bradford protein assay was used to determine soluble protein concentrations in the fermented slurries (Bradford 1976) and samples were compared to a set of bovine serum albumin standard solutions (0.01 to 0.5 mg/mL). Soluble protein data was thus reported as mg of BSA equivalents per g of dry substrate (mg_BSA(eq)_/g_substrate_).

#### *Ulva* sp. carbohydrate content, saccharification products and fermentation metabolites

For the identification of oligosaccharides produced during fermentation, collected supernatants were filtered through 0.2 µm polyethersulfone syringe filters and added into 1.5 mL serum vials. The degree of polymerisation (DP) of produced oligosaccharides was determined using an HPLC system (Agilent, 1200 series) with an Aminex HPX-42A column (300 mm × 7.8 mm, Bio-Rad, California, USA) coupled with a refractive index detector (RID). Twenty µL of sample were injected in the system running isocratic at 0.5 mL/min of ultrapurified water as the mobile phase. Column temperature was kept at 80 °C throughout the analysis. Chromatographic data was initially processed in OpenLab Chemstation (Agilent Technologies LDA, UK). The relative percentage of DP was calculated based on the ratio of each peak area to the total sum of integrated peak areas for each sample. DP identification was performed based on standard malto- and xylooligosaccharide solutions (DP 2–8) (Megazyme Ltd, Ireland).

*Ulva* sp. was also analyzed for total sugar content (soluble and insoluble) following a two-step acid hydrolysis method of the National Renewable Energy Laboratory protocol (NREL/TP-510–42618). Briefly, 300 mg aliquots were hydrolyzed with 72% (v/v) of sulfuric acid at 30 °C for 1 h and then in diluted acid (4%, v/v) at 121 °C for 30 min. After filtration, 20 µL of acid hydrolysates were run through HPLC (Agilent, 1200 series) in an Aminex HPX-87H column (300 mm × 7.8 mm, Bio-Rad, California, USA), operated at 65 °C. Mobile phase consisted of 0.005 M H_2_SO_4_, running at a flow rate of 0.6 mL/min. Quantification of monosaccharides was performed based on external calibration curves of commercially available standards (glucose, arabinose, xylose, galacturonic acid, glucuronic acid, fucose, mannose, and rhamnose) at various concentrations (0.5–10 mg/mL). The same conditions and analytical column were used for the quantification of saccharification products and fermentation metabolites (organic acids) using external standards at concentrations of 0.5–10 mg/mL.

#### Enzymatic activity of *A. oryzae* crude extracts

The enzymatic activity of *A. oryzae*-fermented *Ulva* sp. supernatants was determined according to literature protocols (Wang et al. 2016; Uchida et al. 2019). This involved the mixture of 0.4 mL of crude extract with 1.6 mL of a 1% (w/v) polysaccharide solution (starch, carboxymethyl cellulose (CMC), low-viscosity glucomannan and high-viscosity glucomannan) in 200 mM sodium acetate buffer or 200 mM sodium citrate buffer (both pH 6.0). Mixtures were incubated for up to 240 min at 37 °C, under stir. Reactions were stopped by quenching the mixtures in boiling water for 5 min. Reducing sugars were then measured via the DNS method, adapted from Miller’s original method (Miller 1959). Glucose was used as a standard for the construction of the calibration curve, with concentrations ranging from 0.1 to 1 mg/mL. This data was converted into moles of glucose (m_w_ = 180.156 g/mol) per mL and presented as units of enzymatic activity per mL of fermented slurry. One unit of glycolytic activity (U) was defined as the amount of enzyme capable of releasing 1 µmol of glucose per minute at 37 °C.

Proteolytic activity was determined by mixing 0.2 mL of extract with 1.3 mL of a 1% (w/v) casein solution in 100 mM phosphate buffer (pH 6.8). The mixture was incubated at 37 ºC for 20 min and was then stopped by adding 1.5 mL of a 5% trichloroacetic acid solution. After a centrifugation step (11,600 × *g* for 10 min), the soluble protein content of the supernatant was measured using the Bradford protein assay as previously described. A unit of proteolytic activity was defined as the amount of enzyme capable of releasing 1 µmol of glycine equivalents per minute at the specified incubation conditions.

### Statistical analysis

All analytical methods were performed on either fermentation or control triplicates, with *n* = 3 for every sampling time point. RSM analysis was conducted in Statistica (version 12) (StatSoft Inc., Tulsa, OK, USA). Model assumptions (normality, homoscedasticity, and independence) were inspected via the residual plots. Only linear and quadratic effects were included. One-way analysis of variance (ANOVA) followed by Dunnett’s multiple comparisons test was used for the analysis of the relative abundance of oligo- and monosaccharides. One-way ANOVA followed by Tukey’s multiple comparisons test was used in the comparison of monosaccharide content between the three seaweeds. Student *t*-tests were applied whenever ANOVA was not applicable. These tests, as well as all data plotting, were performed in GraphPad Prism v.6.01 (GraphPad Software, La Jolla, California, USA), with p-value set at 0.05 for all statistical tests.

## Results

### Characterization of *Ulva* sp. as fermentation substrate

Crude protein and carbohydrate analysis of the unprocessed dried *Ulva* sp. used in this study was initially performed, and the results are presented in Table 1.

**Table 1:** Total protein and carbohydrate content of unprocessed *Ulva* sp. and comparison with literature data. All concentrations were determined on a dry weight (w/w) basis (*n* = 3)

		***Ulva ***sp. (present study)	*Ulva *sp. (Bobin-Dubigeon et al. 1997a, b)	*Ulva *sp. (tank farmed) (García-Poza et al. 2022)*	*Ulva *sp. (field harvested) (García-Poza et al. 2022)*
Total protein (g/100 g)		18.49 ± 0.03	11.6	–	–
FAN (mg_Gly(eq)_/g_substrate_)		0.47 ± 0.04	–	–	–
Soluble sugars (mg/g)	Galactose	1.31 ± 0.05	0.4	–	–
	Rhamnose	5.33 ± 0.24	5.8	208.53	133.35
Insoluble sugars (mg/g)	Glucose	97.9 ± 0.1	96.0	345.08**	333.38**
	Xylose	23.0 ± 0.8	37.0	28.10	62.47
	Rhamnose	68.0 ± 2.9	53.0	–	–

*Reference data makes no distinction between soluble and insoluble fractions

**Data reported as the combined concentration of glucose and galactose, with the latter likely to be present in trace amounts

The total protein content of *Ulva* sp. (18.49% w/w) places it in a typical range for several plant-based fermentation substrates. Compared to other staple substrates for food fermentation, it has lower protein content than soybean (35–40% w/w) but considerably more than rice (7% w/w) and wheat (10–14% w/w) (Sanjukta and Rai 2016; Amagliani et al. 2017). When compared to other edible seaweeds, *Ulva* sp. sits in the higher end of Northeast Atlantic edible seaweeds, with its protein content comparing favorably against *F. vesiculosus* (13% w/w) and *Laminaria digitata* (12% w/w) but generally behind that of edible red seaweed such as *P. dioica* (32% w/w) and *Palmaria palmata* (41.5% w/w) (Mæhre et al. 2014). With FAN values around 0.5 mg_Gly(eq)_/g_substrate_, it can be assumed that cultures with limited proteolytic capacity will find this substrate poor in nitrogen. Additionally, *Ulva* sp. contained low amounts of free sugars, with most polysaccharides found in the insoluble fraction. Specifically, acid hydrolysis of insoluble polysaccharides revealed that glucose was the most abundant monosaccharide, followed by rhamnose and xylose. This composition differs from values reported in the literature regarding cell wall-derived carbohydrates in *Ulvales*. Zeroual et al. (2020) reported rhamnose as the most abundant monosaccharide in *Ulva rigida* and *Ulva intestinalis*, followed by xylose, glucuronic acid, glucose, and galactose. This monomer distribution is consistent with the current cell wall models of the *Ulva* genus, highlighting the abundance of the water-soluble ulvan, itself comprised of rhamnose (16.8–45.0%, w/w), xylose (2.1–12.0%, w/w), glucose (0.5–6.4%, w/w), glucuronic acid (6.5–19.0%, w/w), and iduronic acid (1.1–9.1%, w/w), although the range of individual sugars can vary significantly (Wahlström et al. 2020; Li et al. 2023). Cellulose can also contribute up to 15% (w/w) of *Ulva* sp.'s cell wall composition, adding to the amount of glucose in the insoluble fraction (Jackson et al. 2026).

Authors García-Poza et al. (2022) reported on the carbohydrate content of *Ulva* sp. harvested from a location (Figueira da Foz, Portugal) approximately 50 km south of the area where the seaweed of the present study was sourced (Ilhavo, Portugal). The authors list a “galactose + glucose” concentration between 333.38 and 345.08 mg/g, and a rhamnose concentration between 133.35 and 208.53 mg/g. Assuming galactose takes a similarly minor percentage of the monosaccharide profile of their seaweed, their results corroborate the glucose > rhamnose balance verified in our data.

### *Aspergillus oryzae* fermentations of *Ulva* sp.

**Fig. 1:**
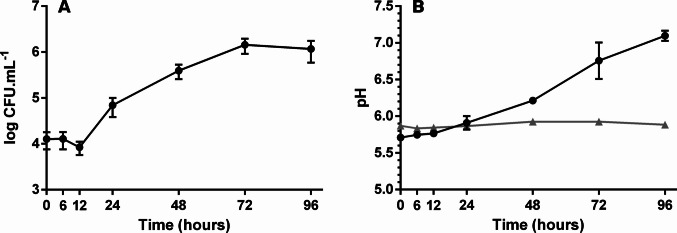
Kinetics of **A** growth of *Aspergillus oryzae* in *Ulva* sp. slurry and **B** concurrent changes in pH. Closed circles correspond to the *A. oryzae* fermentation. Controls, represented by grey closed triangles, consisted of uninoculated media subjected to the same fermentation conditions. Data presented as average ± the standard deviation of three independent cultures (*n* = 3)

Non-saccharified *Ulva* sp. was fermented using *A. oryzae*, both for the assessment of its ability to grow in the algal substrate and for the production of the enzymatic crude extract used in the later saccharifications. Figure 1 shows the growth and pH evolution of *A. oryzae* cultivated in *Ulva* sp. substrate.

A three-fold log increase in viable colony counts of *A. oryzae* on *Ulva* sp. was observed (Fig. 1A), which reached its peak after 72 h and after an initial 12-h lag phase. Further investigation into the metabolites of the *A. oryzae*-fermented *Ulva* sp. revealed minimal changes in FAN, whereas an absence of organic acids was noted during HPLC analysis. Previous studies have achieved comparable growth of other *Aspergillus* strains using *Ulva* as substrate. Fernandes et al. (2019) combined enzymatic extracts of *Aspergillus ibericus*, characterized by high xylanase, cellulase, and β-glucosidase activity, with commercial cellulases for an effective hydrolysis of the glucans present in *Ulva rigida*. Karray et al. (2016), while not quantifying growth directly, processed the same seaweed using *Aspergillus niger* and obtained extracts rich in β-glucosidase, pectinase, and cellulase.

**Fig. 2:**
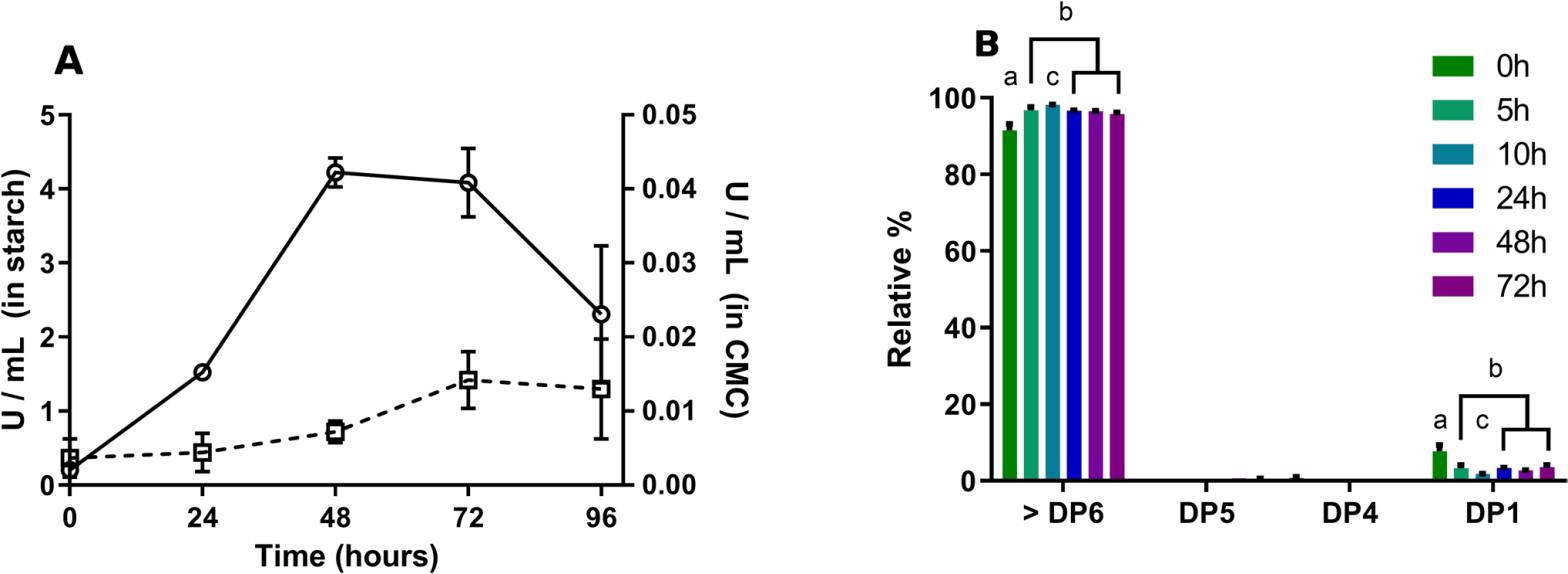
Polysaccharide-degrading activity of *Aspergillus oryzae* on *Ulva* sp. cultures: **A** α-amylase and cellulase and **B** relative abundance of mono- and oligosaccharides in the fermented seaweed slurry. Enzymatic activity was measured against starch (open circles, left Y axis) and carboxymethyl cellulose (CMC) (open squares, right Y axis). Within each DP category, lettering groups statistically non-differentiated results (*p* < 0.05). Data presented as average ± standard deviation of three independent cultures (*n* = 3)

Two approaches were taken to evaluate the capabilities of *A. oryzae* as an algal polysaccharide-degrading culture: (i) a direct measurement of the enzymatic activity during fermentation (Fig. 2A) and (ii) the determination of the degree of polymerization of soluble sugars in the fermented media (Fig. 2B).

Of the four substrates (starch, CMC, low-viscosity glucomannan, and high-viscosity glucomannan) used to test the enzymatic capabilities of *A. oryzae* grown in *Ulva* sp., only starch and CMC yielded quantifiable differences in reducing sugar content. Amylolytic activity was substantially higher than that of cellulose-degrading enzymes, with maximum activity of 4.36 U/mL after 48 h of fermentation. By contrast, the highest activity in CMC was about 0.02 U/mL after 96 h (Fig. 2A), indicating that the crude extract exhibited considerably higher amylolytic rather than cellulolytic potential. Nevertheless, the maximum α-amylase activity measured in *Ulva* sp. cultures was still significantly lower than that reported in starch-dense media (Da et al. 2016; Uchida et al. 2019). It is also noted that the extracts exhibited no quantifiable proteolytic activity on casein.

### Optimization of *Ulva* sp. enzymatic saccharification

**Fig. 3:**
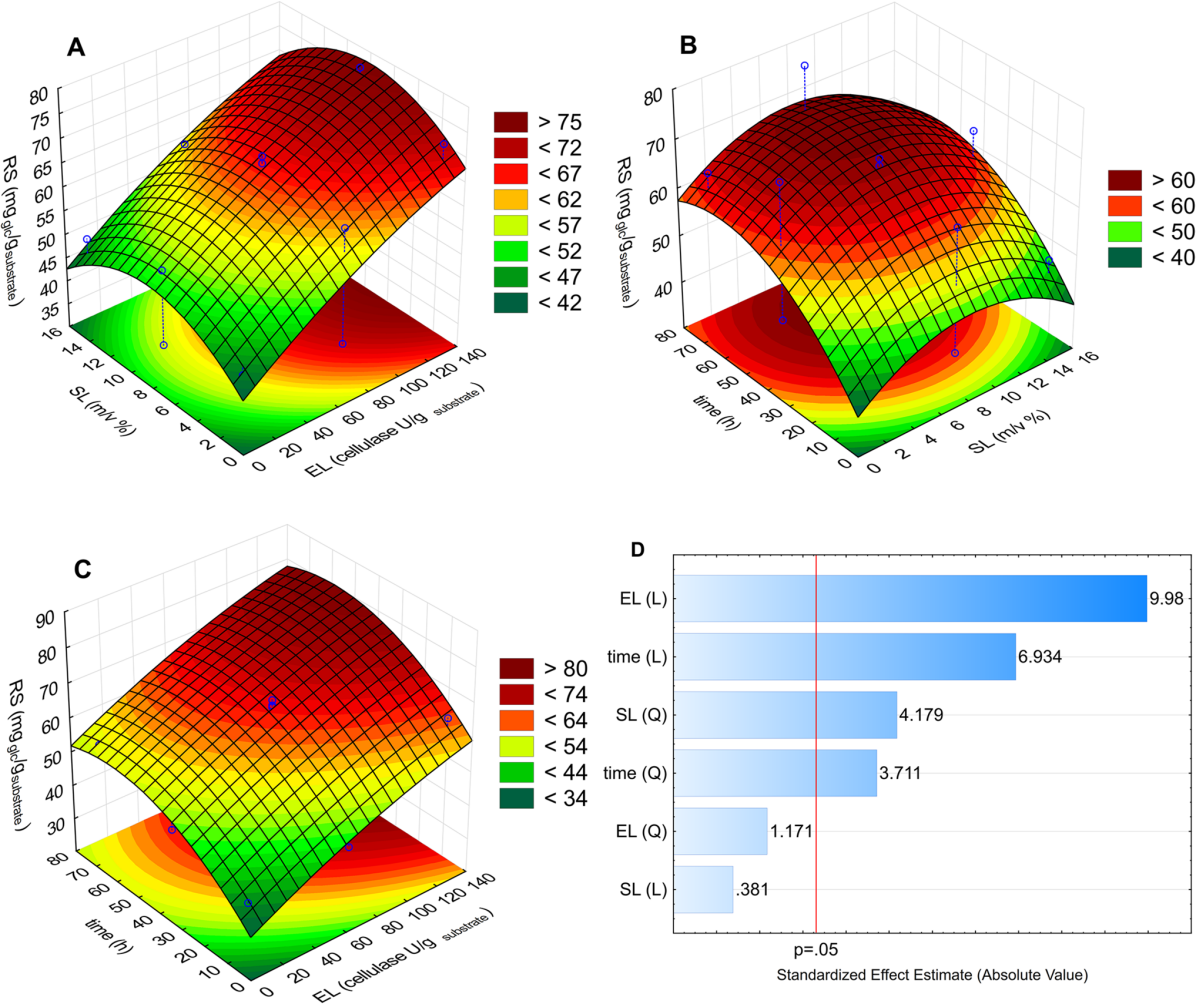
Optimization of *Ulva* sp. saccharification using the combined crude extract and cellulolytic cocktail (CE + CC), represented in three-dimensional surface response plots of the effect of the following: **A** substrate load and enzymatic load; **B** time and substrate load; and **C** time and enzymatic load on the concentration of reducing sugars (RS) (mg_Glu_/g_substrate_). The Pareto analysis chart (**D**) demonstrates the significance, either linear “L” or quadratic “Q”, of each factor

The optimization of *Ulva* sp. saccharification involved the evaluation of three variables, namely substrate load (SL), enzymatic load (EL), and time, using three enzymatic mixtures: that of *A. oryzae* crude extract (CE), a commercial enzymatic cocktail (CC), and a combination of the two (CE + CC). The graphical representation of the latter (CE + CC) can be seen in Fig. 3. The effect of the tested variables on reducing sugar yields was not drastically different across enzymatic sources; the contour plots and Pareto charts of the first two sources (CE and CC) can be found in supplementary material (Figures S2 and S3). Equations and goodness-of-fit statistical parameters for the three dependent variables for all optimizations are provided in supplementary table S3.

The maximum concentration of reducing sugars was around 78 mg_Glu_/g_substrate_ and was obtained in the highest EL and reaction times, at a SL of 8% (w/v). Supplementary figures S1 and S2 display the optimization data for CE and CC tested in isolation. Notably, their maximum yields [18.6 mg/g (CE) and 63.5 mg/g (CC)] closely add up to the value found in the combined extract [78 mg/g (CE + CC)]. Higher enzymatic loads provided greater reducing sugar concentrations. Longer reaction times are likely to further benefit the hydrolysis, though the plateauing of reducing sugar yields after 48 h suggests diminishing returns. A theoretical peak was achieved at the 55.5 h mark. Substrate load yielded maximum returns near the midpoint of the test range at 8% (w/v). Having achieved the highest reducing sugar concentration under these conditions, an additional batch of *Ulva* sp. was saccharified using the combined crude extract and cellulolytic cocktail (CE + CC) with a substrate load of 8% (w/v), enzymatic load of 133 U/g_substrate_, and reaction time of 55.5 h. Hydrolysates were subsequently used in batch flask fermentations using *L. plantarum* and *C. jadinii*, whereas non-saccharified cultures were used as control.

### Carbohydrate profile of saccharified *Ulva* sp.

The monosaccharide profile of *Ulva* sp. saccharified according to the abovementioned optimized conditions, as well as those obtained for the individual enzymatic sources [*A. oryzae* extract (CE), cellulolytic cocktail (CC)] at their respective optimums, are presented in Table 2.

**Table 2:** Monosaccharide content of the aqueous fractions of saccharified *Ulva* sp. Samples were obtained from the same high-yielding condition of the RSM optimization (time = 48 h, SL = 8% w/v, EL = 133 mg/g) for all enzymatic treatments: *CE*, crude extract; *CC*, cellulolytic cocktail; and *CE + CC*, extract and cocktail mix

		**CE**	**CC**	**CE + CC**
Soluble sugars (mg/g)	Glucose	45.92 ± 6.12 (a)	85.31 ± 1.47 (b)	157.08 ± 1.07 (c)
	Galactose	21.88 ± 2.65 (a)	10.66 ± 2.32 (b)	25.63 ± 0.60 (a)
	Rhamnose	–	1.27 ± 0.22 (a)	1.11 ± 0.10 (a)
	Galacturonic acid	233.92 ± 24.82 (a)	151.07 ± 4.40 (b)	153.83 ± 1.43 (b)

Values are given as average ± standard deviation (*n *= 3). Different letters within a row indicate significant differences (*p* < 0.05). Statistical analysis was performed using one-way ANOVA followed by Tukey’s post hoc test (glucose, galactose, galacturonic acid) or Student’s *t*-test (rhamnose)

Glucose concentrations achieved in the combined set [157.08 mg/g (CE + CC)] roughly equated the sum of the glucose concentrations obtained with each of its individual constituent extracts. The same effect was not present for any of the other detected monosaccharides. Few traces of rhamnose, xylose, and glucuronic acid, the main detectable monomeric constituents of ulvan, were identified, hinting at a minimal breakdown of the major cell wall polysaccharide. The highest concentration of any individual monosaccharide detected was that of galacturonic acid after hydrolysis by the *A. oryzae* crude extract (CE), reaching 233.92 mg/g. This concentration was not matched by the optimized CE + CC saccharification (153.83 mg/g) despite equal concentrations of *A. oryzae* extract.

Comparisons of the enzymatic treatment applied in this study with published literature give an overall favorable view of the results achieved. Treatments of heat-processed *Ulva* sp. using Celluclast alone, such as those conducted by Yahmed et al. (2025), reach glucose yields (approximately 53 mg/g) below those obtained in CC (85.31 mg/g), reinforcing the benefits of using β-glucosidase. A more optimized approach by Jackson et al. (2026) involving a mix of endo- and exo-cellulases recovered a maximum of 188 mg/g dw, narrowly surpassing the CE + CC treatment, although no information on the nature of the recovered sugars was provided.

**Fig. 4:**
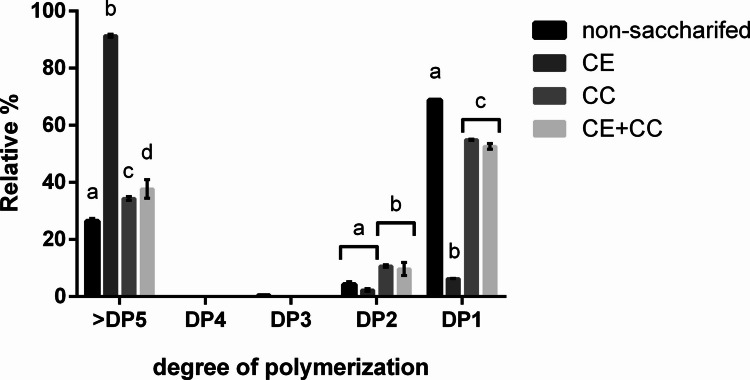
Relative abundance of oligo- and monosaccharides in the soluble fraction of the saccharified *Ulva* sp. Samples were obtained from the same high-yielding condition of the RSM optimization (time = 48 h, SL = 8% w/v, EL = 133 mg/g) for all enzymatic treatments: CE, crude extract; CC, cellulolytic cocktail; and CE + CC, extract and cocktail mix. Within each DP category, lettering groups statistically non-differentiated results (*p* < 0.05). Data presented as average ± standard deviation of three replicas of the aforementioned saccharification conditions (*n* = 3)

Figure 4 shows a qualitative analysis of oligo- and monosaccharides (shown as relative percentage) in the optimization runs yielding the highest reducing sugar content, sorted by degree of polymerization (DP).

The majority of oligosaccharides in *A. oryzae* extracts (CE) were of DP of 5 or higher, suggesting a higher ratio of endo- to exolytic activity, not seen in the other enzymatic mixtures (CC and CC + CE). These, in contrast, exhibited DP1 as the highest fragment size in proportion, likely due to the presence of the exolytic β-glucosidase. DP1-sized residues equally dominated the relative distribution of fragments in the non-saccharified *Ulva* sp., though this is likely a reflection of the limited amount of extractable oligosaccharides.

### Evaluation of saccharified *Ulva* sp. as substrate for microbial fermentations

#### *L. plantarum* fermentations

**Fig. 5:**
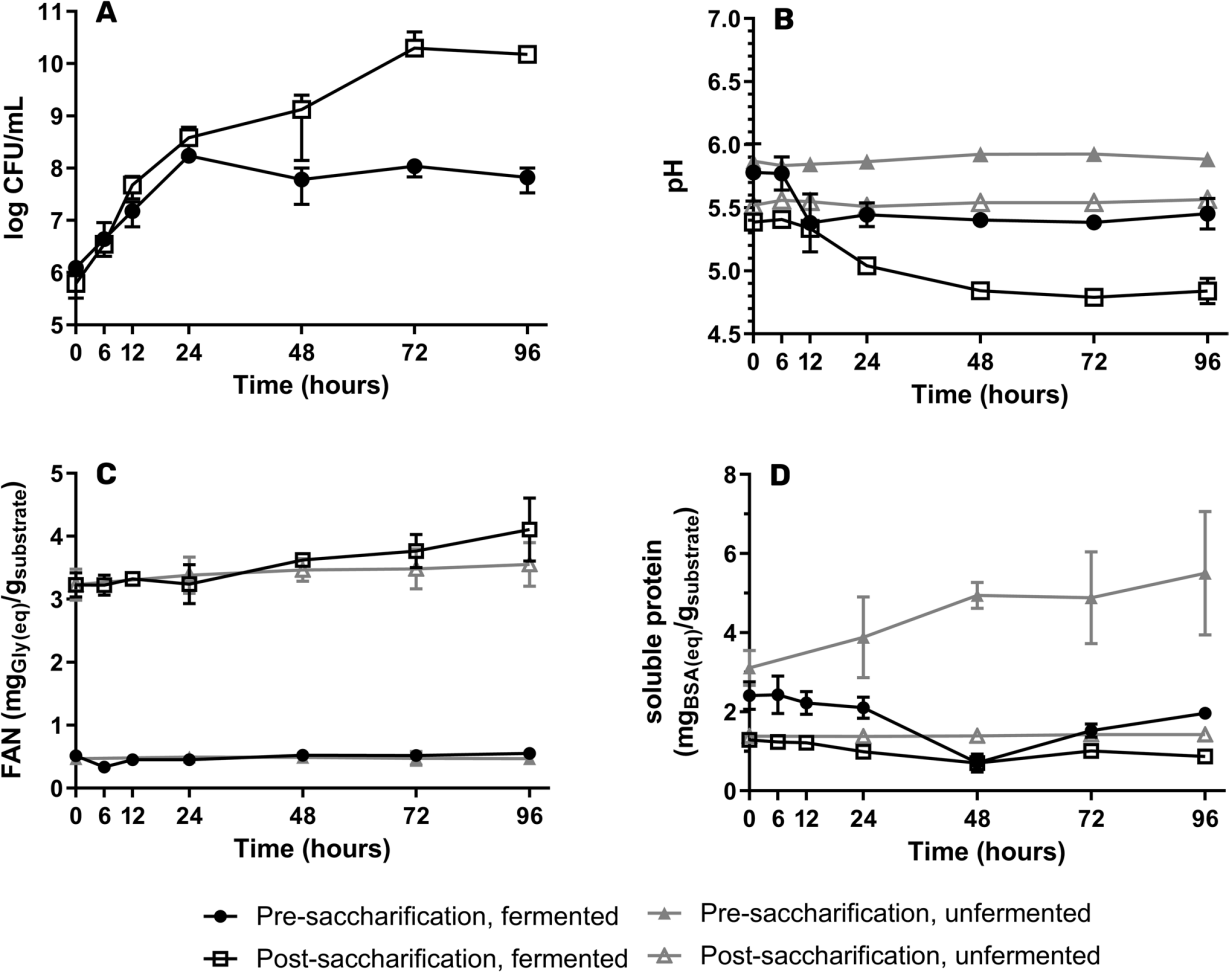
Pre-saccharification (closed circles) and post-saccharification (open squares) fermentation of *Ulva* sp. by *L. plantarum* and changes in **A** viable colony counts (log colony forming units per ml of fermentation media), **B** pH, **C** free amino nitrogen (FAN) (mg of glycine equivalents per g of dry seaweed), and **D** total soluble protein (mg of BSA equivalents per g of dry seaweed). Non-saccharified unfermented controls are represented by faded closed triangles. Their saccharified counterparts are represented by open faded triangles. Data presented as average ± the standard deviation of three independent cultures (*n* = 3)

The fermentation of native and saccharified *Ulva* sp. by *L. plantarum* was evaluated, as depicted in Fig. 5.

The use of saccharified *Ulva* sp. as a fermentation medium for *L. plantarum* led to substantial improvements in all monitored growth parameters. Viable colony counts (Fig. 5A) reached a maximum of over 10 log CFU/mL after 72 h of fermentation (*µ *= 0.14 h^−1^), as opposed to the non-saccharified cultures, which reached a peak of 8 log CFU/mL at 24 h and then exhibited a declining trend, indicating loss of viability. This is likely attributed to the low quantities of assimilable sugars found in *Ulva* sp. Figure 5C shows that the concentration of FAN was enhanced by saccharification, from an average of 0.52 mg/g_substrate_ to approximately 3.2 mg/g_substrate_. Given the absence of proteolytic activity from the CE, this increase is the expected outcome of alcalase being part of the commercial enzymes employed. From this enhanced baseline, concentrations slowly rose in the saccharified fermentation to a maximum of 4.1 mg/g_substrate_ after 96 h, reverting the pattern observed for the non-saccharified fermentation. This implies a degree of proteolytic activity by *L. plantarum*, capable of offsetting its own consumption of amine nitrogen present in the media.

**Fig. 6:**
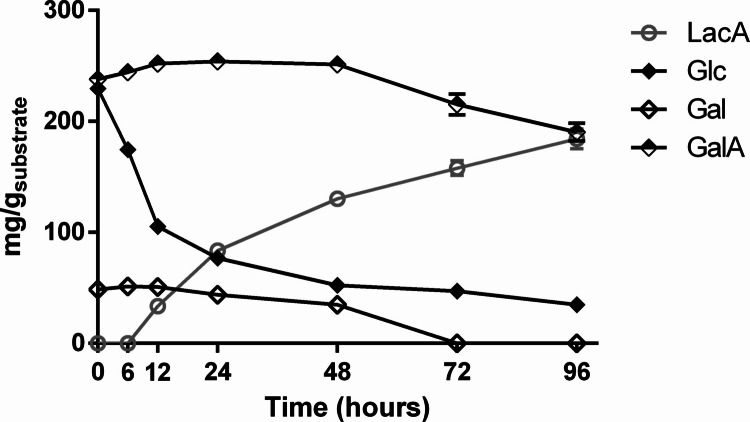
Concentration of soluble sugars [glucose (Glc), galactose (Gal), and galacturonic acid (GalA)] and metabolites [lactic acid (LacA)] during fermentation of saccharified *Ulva* sp. by *Lactiplantibacillus plantarum*. Data presented in mg per g of dry substrate (mg/g_substrate_) and is the average ± the standard deviation of three independent cultures (*n* = 3)

Figure 6 depicts the concentration of soluble sugars and organic acids in the fermentation media. The production of lactic acid, a predominant feature of LAB, is the most likely cause for the acidification seen in Fig. 5B, greatly intensified by the saccharification process.

Lactic acid was the only organic acid detected in the analysis performed and is likely responsible for the acidification observed in Fig. 5B. Figure 6 also shows the consumption of different carbon sources over time, with glucose levels rapidly decreasing during the first 24 h of fermentation and galactose steadily depleting thereafter.

#### *C. jadinii* fermentations

**Fig. 7:**
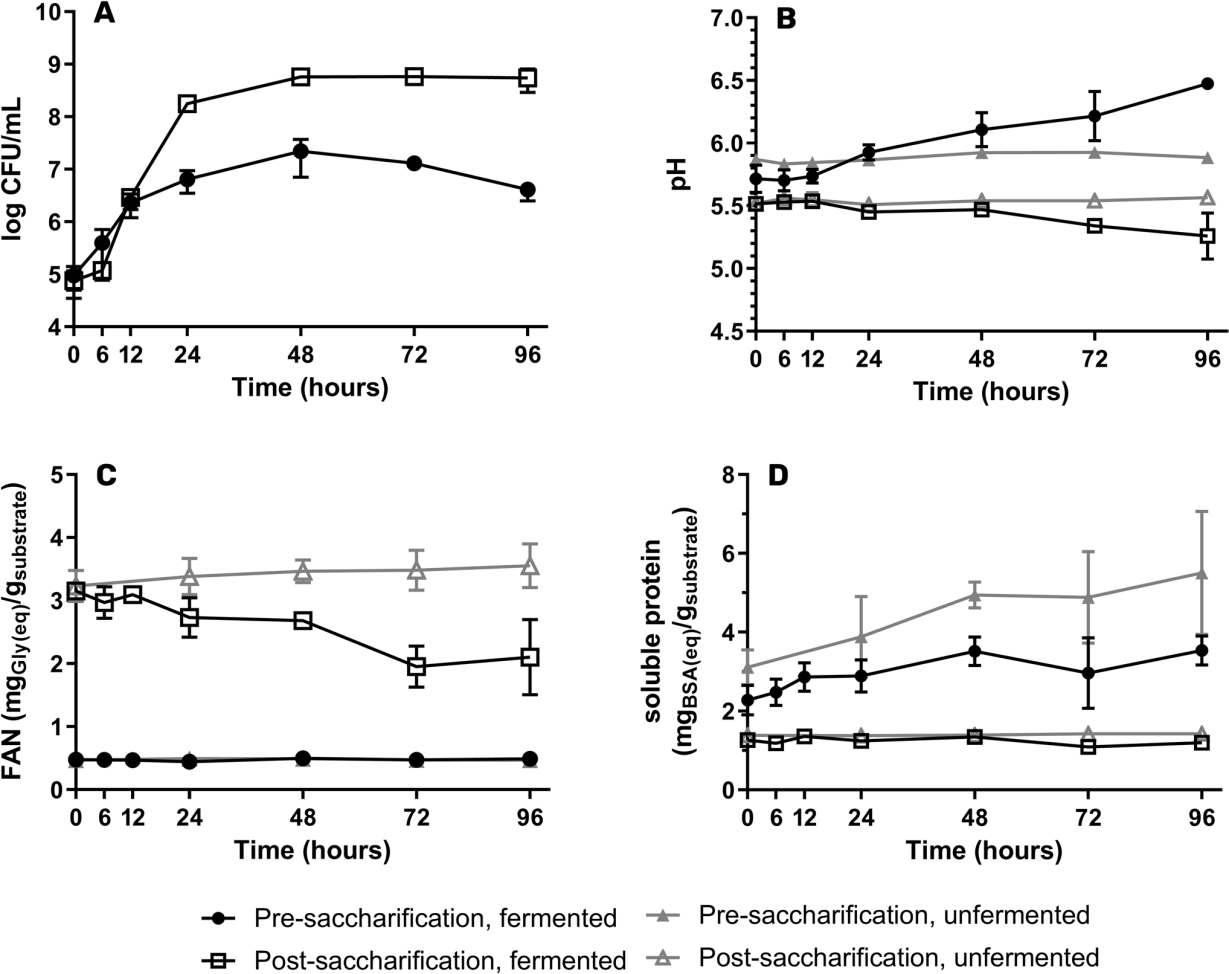
Pre-saccharification (closed circles) and post-saccharification (open squares) fermentation of *Ulva* sp. by *C. jadinii* and the monitored changes in the following: **A** viable colony counts (log colony forming units per ml of fermentation media); **B** pH; **C** free amino nitrogen (FAN) (mg of glycine equivalents per g of dry seaweed); and **D** total soluble protein (mg of BSA equivalents per g of dry seaweed). Non-saccharified unfermented controls are represented by faded closed triangles. Their saccharified counterparts are represented by open faded triangles. Data presented as average ± the standard deviation of three experimental replicas performed simultaneously (*n* = 3)

Figure 7 depicts the impact of the optimized saccharification of *Ulva* sp. on the growth of *C. jadinii*.

Major distinctions between the treatments are noticeable after 24 h, with a difference in CFU/mL of approximately two log cycles. This difference widened as viability in the non-saccharified fermentation began to fall between 48 and 72 h. Both fermentations reached a stationary phase at similar time points, suggesting that nutrient depletion occurred at the same time, despite different rates of intake. This may, in turn, suggest that the fermentation is still constrained by a lack of assimilable sugars.

**Fig. 8:**
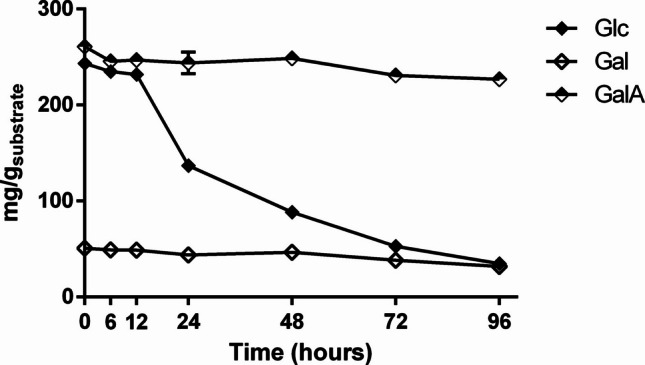
Concentration of soluble sugars [glucose (Glc), galactose (Gal), and galacturonic acid (GalA)] during the fermentation of saccharified *Ulva* sp. by *Cyberlindnera jadinii*. Data presented in mg per g of dry substrate (mg/g_substrate_) and is the average ± the standard deviation of three independent cultures (*n* = 3)

The monitoring of monosaccharides depicted in Fig. 8 presents glucose as the primary energy source for *C. jadinii*. After a steep consumption during the initial 24 h, its intake gradually slowed down.

Glucose is presented as the primary energy source for *C. jadinii* and after a steep consumption in the first 24 h, mirrored by a steep increase in cell counts (Fig. 5A), its intake gradually slowed down. Early studies on the substrate preferences of this yeast reported a slow intake of galactose due to carbon catabolite repression. This intake accelerates significantly once glucose is fully consumed and is on par with the yeast’s ability to assimilate xylose and mannose (Šestáková 1979; Martins et al. 2020). However, unlike *L. plantarum, C. jadinii* did not show signs of substantial intake of alternative sugars. Changes in pH during fermentation followed different tendencies between the two treatments, with the pre-saccharification increase in pH being replaced by a slight acidification. Given that no organic acids were detected in the HPLC analysis, this change is likely related to a reduced rate of alkaline products being released. Figure 7C also shows that, in the saccharified seaweed, FAN concentrations steadily decreased as the fermentation progressed. This suggests a steady consumption of nitrogen without the peptide breakdown-related release seen in the media fermented by *L. plantarum*. This observation is near universal in cases where *C. jadinii* ferments media devoid of harsh inhibitory factors and is an indicator of an overall improvement in the quality of the substrate (Buerth et al. 2016; Lapeña et al. 2020).

## Discussion

The structure and composition of seaweed cell walls can be an obstacle for most traditional microbial food cultures (Uchida and Miyoshi 2013; Maneein et al. 2018). Most substrate pre-treatment approaches and the various aspects of the biochemistry involved are underexplored (Ren et al. 2025). The compositional analysis performed paints *Ulva* sp. as a poor substrate for microbial fermentations due to its limited amounts of assimilable sugars and FAN. Assuming these properties are shared among other intertidal seaweed species, they constitute a possible explanation for their limited adoption as substrates in food fermentation processes. Fungal strains of the *Aspergillus* genus are known producers of extracellular enzymes, including a range of cellulases, amylases, pectinases, proteases, xylanases, and many others (Matsuzawa et al. 2020; Daba et al. 2021). *A. oryzae* is especially well equipped to break down plant starch, as it is capable of releasing α-amylase, α-glucosidase, and glucoamylases in the presence of either starch or its related oligosaccharides, including maltose and dextrin (Da et al. 2016). On this basis, *A. oryzae* fermentations of *Ulva* sp. were conducted, with the intent of producing an enzymatic extract capable of hydrolysing the cell wall of the green seaweed. Its effectiveness was compared to—and supplemented by—a cellulolytic cocktail of commercially-acquired enzymes.

A synergistic interaction between the *A. oryzae* crude extract (CE) and the cellulolytic cocktail (CC) was revealed when these enzymatic sources were combined in an optimized saccharification of *Ulva* sp. Having achieved a final concentration of 157 mg/g of glucose equivalents, it is likely that an extensive breakdown of cellulose occurred. The results also suggest that the CE may have played a larger role in *Ulva* sp. saccharification as a contributor to the release of galactose and uronic acids. An endo-cellulolytic enzymatic activity is likely responsible for the near absolute abundance of oligosaccharide fragments sized DP5 and above in the exclusively fungi-saccharified seaweed. This same mechanism could explain the glucose concentrations in the combined mixture sitting well above those obtained using exclusively commercial enzymes. Determining the specific type of enzymatic activity present in the CE, and how it differs from the cellulolytic enzymes used can reveal valuable insights on how to further enhance the saccharification of seaweed substrates. Throughout this process, the amount of alcalase, initially added to assist in the breakdown of peptides and the release of assimilable nitrogen, was kept constant. While this approach limited the study of protein-polysaccharide interactions, it opens the possibility for further enzymatic optimizations in the future.

The absence of significant amounts of rhamnose, xylose, and glucuronic acid suggested that ulvan was left intact by all methods of saccharification. Such result is not entirely unexpected, given that most lyases specialized in ulvan breakdown have been recovered from marine microbial sources (Qiao et al. 2020). The presence of xyloglucans has also been reported in most categorizations of the cell wall of *Ulva* sp., making the complete absence of xylose in these results further evidence that these saccharifications were, most likely, centered around cellulose or starch (Wahlström et al. 2020; Li et al. 2023). Further optimizations can involve the hydrolysis of *Ulva*-specific sulfated polysaccharides in order to recover a wider variety of fermentable sugars. Enzymatic approaches to such task will require a deeper understanding of the chemistry of seaweed cell walls, as well as the use of highly specialized hydrolases.

*L. plantarum* is a versatile bacterial species, but its limitations in extracting fermentable sugars from sources of insoluble fiber have been previously documented (Scheirlinck et al. 1990). Previous attempts at using this microorganism in seaweed fermentation have resorted to enzymatic pre-treatments targeting the pre-emptive breakdown of cell walls (Zabidi et al. 2020; Lin et al. 2020). Similarly, *C. jadinii* is regarded as an adaptable microorganism with wide substrate compatibility, but its use in the fermentation of cellulose-rich substrates is often paired with chemical or enzymatic pre-digestions (Buerth et al. 2016; Sousa-Silva et al. 2021). The chosen combination of enzymatic extracts managed to significantly improve the growth of *L. plantarum*, which was reflected in an increase in viable colony counts, greater release of FAN, and greater media acidification. These increases were likely caused by the greater availability of carbon sources, as the bacteria consumed most of glucose in the fermentation media and then resorted to alternative sugars, absent from the non-saccharified seaweed. This was also reflected on what appeared to be a diauxic growth curve, with a second growth phase between 48 and 72 h of fermentation. The production of organic acids and consequent changes in pH and organoleptic traits are highly desirable in fermented food products (Redway and Combet 2023). This is especially the case with edible seaweeds, which suffer from low acceptability in Western markets and are highly perishable once removed from their environment (Figueroa et al. 2021). Growth of *C. jadinii* was also enhanced by saccharification, likely due to increased glucose and FAN concentrations. Its additional nitrogen intake, hinted at by the gradual decrease in FAN during fermentation, suggests upregulation of the metabolic pathways that support the synthesis of secondary metabolites, including bioactive and flavor-active compounds (Park and Kim 2020; Liu et al. 2023; Cao et al. 2023). *C. jadinii* is a known producer of glutathione, especially under conditions of nutrient excess (Rai et al. 2019). Its release can lead to a substantial increase in the antioxidant activity of the fermented product, further adding to potential benefits of its application in food and nutraceuticals (Charoensiddhi et al. 2017; Rai et al. 2019). While the impact of growth inhibitors was not assessed in this study, it is likely that the heat processing done to minimize the risk of contamination led to the formation of furfural and HMF. Other inhibitors common in seaweed substrates, such as phlorotannins, bromophenols and flavonoids, are mostly absent from *Ulva* sp. (Wekre et al. 2019).

Lactic acid is a valuable product of LAB fermentations, with applications across many fields of industry including, but not limited to, biodegradable polymers, food additives and preservatives, and as a disinfectant in cleaning and cosmetic products (Ayivi et al. 2020; Abedin et al. 2024). This desirability has led to a search for sustainable substrates, including seaweeds (Lin et al. 2020; Sudhakar and Dharani 2022). Similarly, the production of *C. jadinii* biomass is one of its most common applications, particularly due to its use as feedstock, prompting attempts to maximize this output using alternative substrates (Sousa-Silva et al. 2021; Wu et al. 2025). While saccharified *Ulva* sp. can sustain the growth of both *L. plantarum* and *C. jadinii* in the conditions tested, it is unlikely that the approach taken in this study would ever maximize the yield of lactic acid or yeast biomass. Higher fermentable sugar contents, and thus higher yield potentials, have been achieved in *Ulva rigida* using combinations of chemical and enzymatic hydrolysis (Brandão et al. 2025). Nevertheless, the organoleptic and nutritional transformations induced in the seaweed by the growth of these organisms can make *Ulva* sp. itself a more desirable and stable food resource, building a pathway to its sustainable development.

Regarding sustainability and scalability concerns, this study paired fermentation with accessible and relatively affordable cellulolytic enzymes. As an industrial bioprocess, fermentation is a low emission, low resource-intensive approach to biotransformation, proven to be scalable and compatible with circular bioeconomic policies (Abbaspour 2024). Alternative routes to the implementation of fermented *Ulva* sp. could attempt a more thorough hydrolysis, either through mixed methods or a specialized enzymatic approach using ulvan-lyases, such as those carried out by (Qiao et al. 2020). Such an approach could maximize the recovery of fermentable sugars while minimizing potential inhibitors, greatly transforming the substrate and the products of fermentation. However, the use of enzymes specialized in the breakdown of marine heteropolysaccharides is still largely unaffordable at large scales, only recently being considered for larger scale bioprospecting endeavors (Tang et al. 2021; Jackson et al. 2026).

In conclusion, while seaweeds hold great potential as fermentation substrates, the structure and composition of their cell wall can be an obstacle for the most common microbial food cultures. The effect enzymatic saccharification has on these fermentations is underexplored, and the various aspects of the biochemistry involved are not yet fully known. This study takes a novel approach by combining an enzymatic extract from *A. oryzae* and a set of commercial cellulolytic enzymes and managed to successfully improve the growth of both LAB (*L. plantarum*) and a yeast (*C. jadinii*) on *Ulva* sp. substrate. Relevant fermentation parameters such as the acidification and FAN release under *L. plantarum* were improved, and the additional intake of nitrogen by *C. jadinii* suggests an upregulation of secondary metabolism pathways deserving of further study. Based on the obtained reducing sugar yields, only partial breakdown of *Ulva* sp. cell wall was achieved; further optimisation should involve the hydrolysis of *Ulva*-specific sulfated polysaccharides such as ulvan. Enzymatic approaches to such task will require a deeper understanding of the chemistry of seaweed cell walls, as well as the use of highly specialized hydrolases.

## Supplementary Information

Below is the link to the electronic supplementary material.

ESM 1 (PDF 1.36 MB)

## Data Availability

The raw data collected for this study has not yet been made available in a public repository but can be shared with permission from the corresponding authors.
