# Supplementary material for: Effect of enzymatic saccharification on edible seaweed (Ulva sp.) fermentation
Source: Appl Microbiol Biotechnol. 2026 May 28;110:225. doi: 10.1007/s00253-026-13864-4 (PMC13407968; doi:10.1007/s00253-026-13864-4)
Supplement: Supplementary file 1 [file 253_2026_13864_MOESM1_ESM.pdf]

Supplementary Material

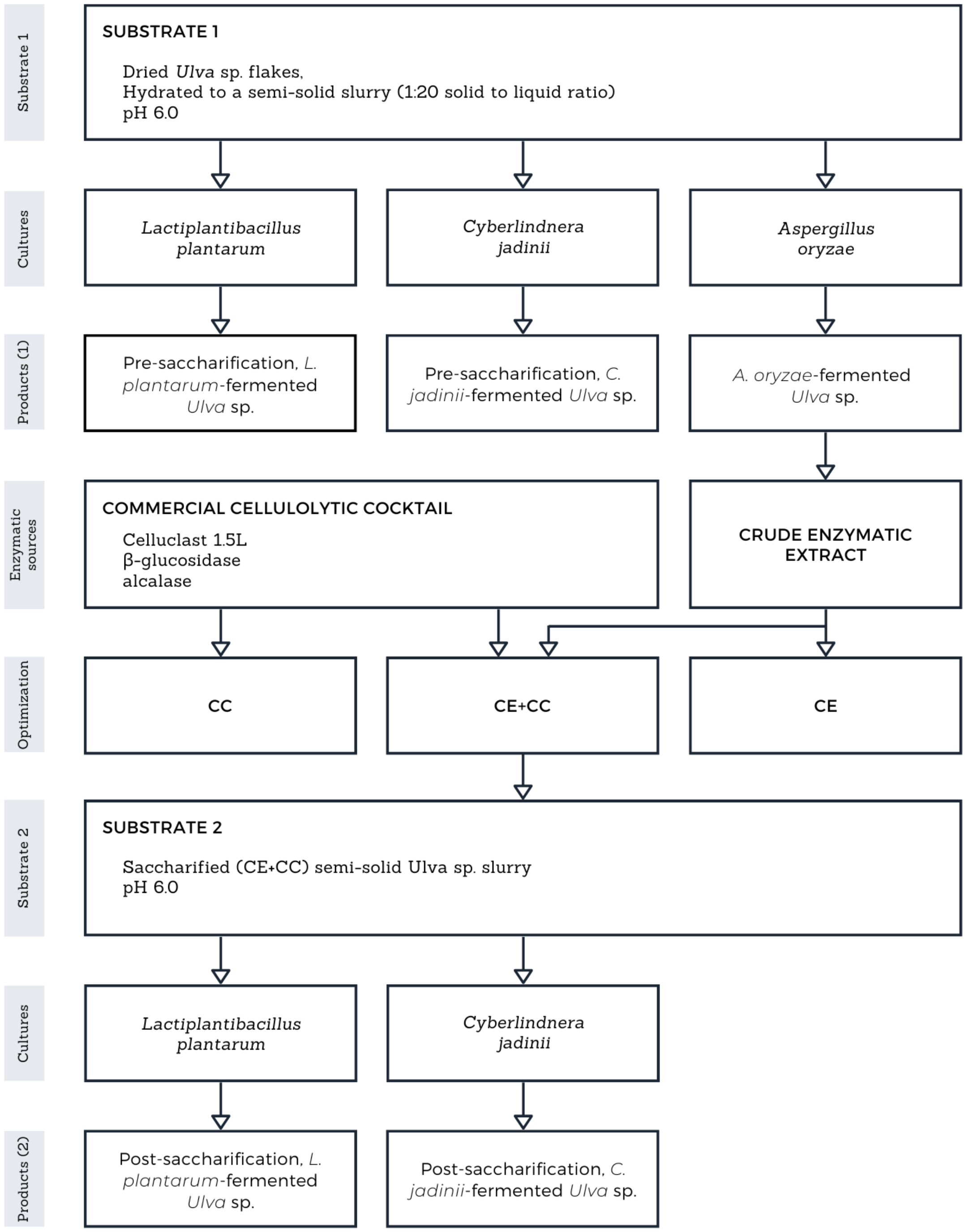

Figure S1 Flowchart representation of the experimental design employed in this study. Substrate 1 is the non-saccharified *Ulva* sp. semi-solid fermentation media, used to obtain the pre-saccharification data displayed in Figures 5 to 9 of the main manuscript, as well as the crude enzymatic extract from *Aspergillus oryzae*. Labels CC (commercial cellulolytic cocktail), CE (crude extract from *A. oryzae* fermentation) and CE+CC (combination of both previous extracts) correspond to the enzymatic sources subjected to response surface methodology optimization. Of the three sources, CE+CC was used in the follow-up saccharifications of *Ulva* sp., producing Substrate 2 (saccharified *Ulva* sp.). Fermentation of this substrate by *Lactiplantibacillus plantarum* and *Cyberlindnera jadinii* yielded the post-saccharification data of Figures 5 to 9.

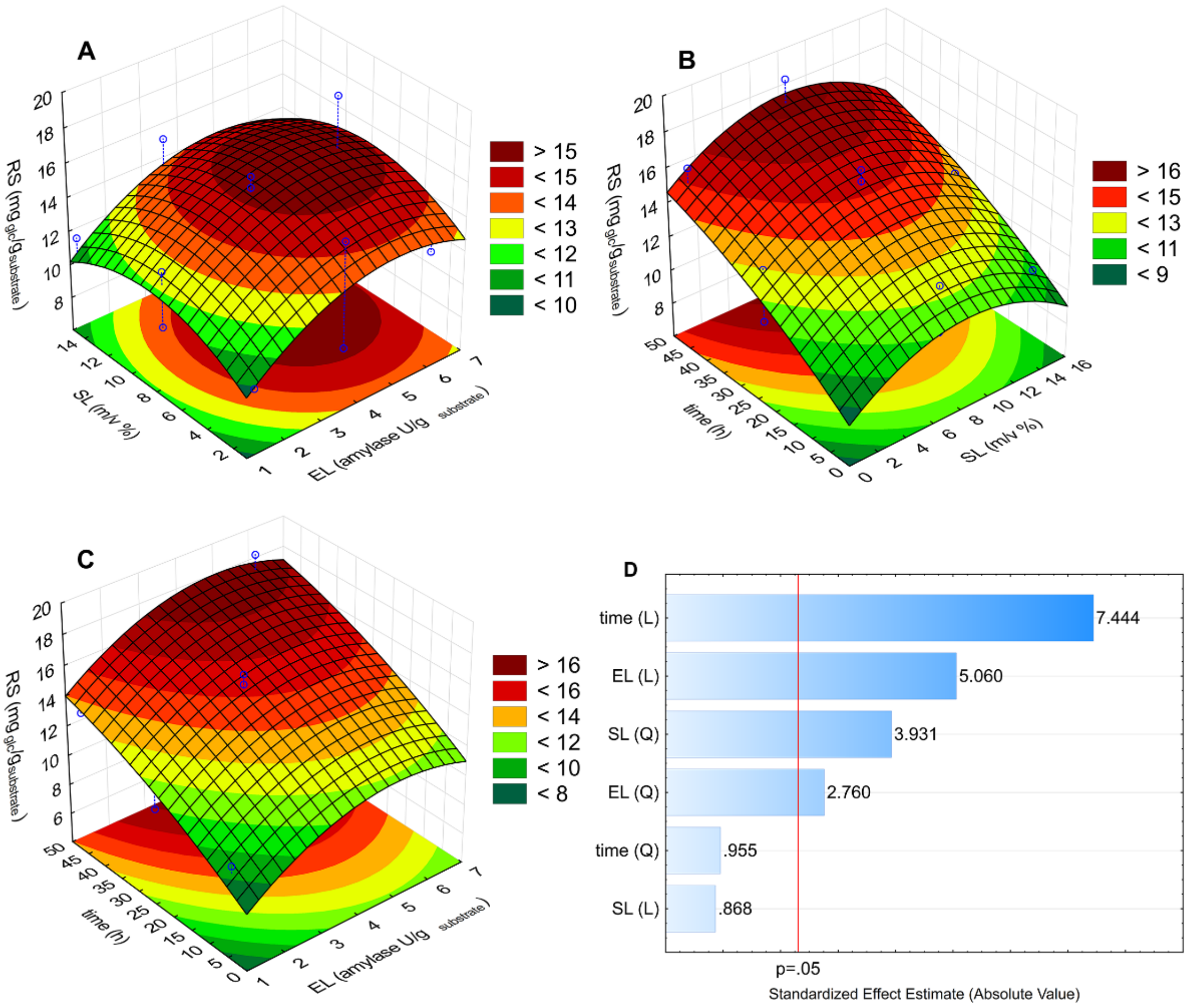

Figure S2 Optimization of *Ulva* sp. saccharification using *Aspergillus oryzae*-fermented *Ulva* sp. extract (CE), represented in three-dimensional surface response plots of the effect of (A) substrate load and enzymatic load, (B) time and substrate load, and (C) time and enzymatic load on the concentration of reducing sugars (RS) ($mg_{Glu}/g_{substrate}$). The Pareto analysis chart (D) demonstrates the significance, either linear "L" or quadratic "Q", of each factor.

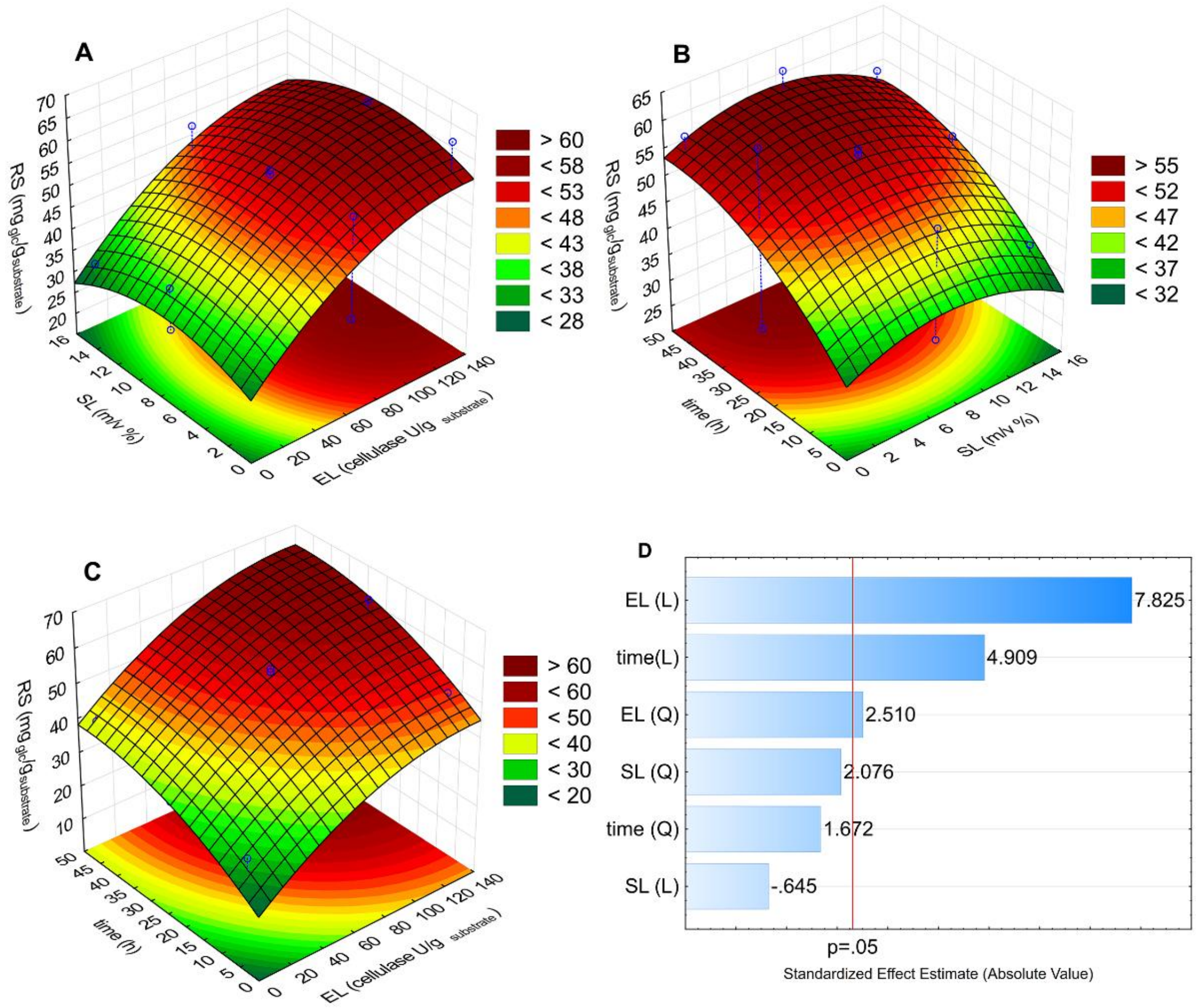

Figure S3 Optimization of *Ulva* sp. saccharification using the cellulolytic enzymatic cocktail (CC), represented in three-dimensional surface response plots of the effect of (A) substrate load and enzymatic load, (B) time and substrate load, and (C) time and enzymatic load on the concentration of reducing sugars (RS) ($mg_{Glu}/g_{substrate}$). The Pareto analysis chart (D) demonstrates the significance, either linear "L" or quadratic "Q", of each factor.

Table S1 Independent variables (factors) and their levels used in the RSM optimization of the enzymatic saccharification of *Ulva sp*. using i) crude extract from the *A. oryzae* fermentation (CE), ii) a commercial cellulolytic cocktail (CC), and iii) the combination of both (CE+CC). Factors included substrate load (SL), enzymatic load (EL), and reaction time. Sets were performed sequentially, with modifications performed in between highlighted in bold.

		Factor levels		
		Minimum	Midpoint	Maximum
Set 1 (CE)	SL (m/v %)	1	8	15
	EL (amylase U/g $_{substrate}$)	1.2	3.6	6
	Time (h)	6	27	48
Set 2 (CC)	SL (m/v %)	1	8	15
	**EL (cellulase U/g $_{substrate}$)**	**7**	**70**	**133**
	Time (h)	6	27	48
Set 3 (CE+CC)	SL (m/v %)	1	8	15
	EL (cellulase U/g $_{substrate}$)	7	70	133
	**Time (h)**	**6**	**39**	**72**

Table S2 Design of experiment for Set 3 (CE+CC) of the RSM optimization of the enzymatic saccharification of *Ulva* sp. (EL) enzymatic load is represented in cellulase U/g substrate, (SL) substrate load is represented as % (m/v), time is represented in hours.

Run Nr	EL	SL	Time	Cellulase (uL)	β-glucosidase (uL)	Alcalase (mL)	Aspergillus extract (mL)	Buffer (mL)	Seaweed mass (g)
1	7	1	27	5	0.5	0.3	0.1	49.6	0.5
2	133	1	27	95	9.5	0.3	0.5	49.1	0.5
3	7	15	27	75	7.5	4.43	1.5	44	7.5
4	133	15	27	1,425	142.5	4.43	7.5	36.5	7.5
5	7	8	6	40	4	2.33	0.8	46.8	4
6	133	8	6	760	76	2.33	4	42.8	4
7	7	8	48	40	4	2.33	00.8	46.8	4
8	133	8	48	760	76	2.33	4	42.8	4
9	70	1	6	50	5	0.3	0.3	49.3	0.5
10	70	15	6	750	75	4.43	4.5	40.2	7.5
11	70	1	48	50	5	0.3	0.3	49.3	0.5
12	70	15	48	750	75	4.43	4.5	40.2	7.5
13	70	8	27	400	40	2.33	2.4	44.8	4
14	70	8	27	400	40	2.33	2.4	44.8	4
15	70	8	27	400	40	2.33	2.4	44.8	4

Table S3 Equations and goodness-of-fit statistical parameters for the modelling of reducing sugar concentration (RS)($mg_{glc}/g_{substrate}$) as a function of the tested factors (enzymatic load (EL), substrate load (SL) and time (t)) for each extract. Statistical parameters presented are the overall correlation coefficient (R^2) and *p*-value.

Extract	Equations	R^2	*p*-value
CE	$\Delta RS = 6.757 + 2.415\ (EL) - 0.237\ (EL^2) + 0.677\ (SL) - 0.040\ (SL^2)$<br>$\Delta RS = 8.383 + 0.677\ (SL) - 0.040\ (SL^2) - 0.177\ (t) - 0.001\ (t^2)$<br>$\Delta RS = 5.633 + 2.415\ (EL) - 0.237\ (EL^2) - 0.177\ (t) - 0.001\ (t^2)$	0.9283	0.0852
CC	$\Delta RS = 29.453 + 0.385\ (EL) - 0.001\ (EL^2) + 1.370\ (SL) - 0.094\ (SL^2)$<br>$\Delta RS = 33.802 + 1.370\ (SL) - 0.094\ (SL^2) + 0.809\ (t) - 0.008\ (t^2)$<br>$\Delta RS = 18.161 + 0.385\ (EL) - 0.001\ (EL^2) + 0.809\ (t) - 0.008\ (t^2)$	0.9241	0.0231
CE+CC	$\Delta RS = 41.505 + 0.259\ (EL) - 0.001\ (EL^2) + 2.438\ (SL) - 0.148\ (SL^2)$<br>$\Delta RS = 38.418 + 2.438\ (SL) - 0.148\ (SL^2) + 0.711\ (t) - 0.006\ (t^2)$<br>$\Delta RS = 32.813 + 0.259\ (EL) - 0.001\ (EL^2) + 0.711\ (t) - 0.006\ (t^2)$	0.9568	0.0002
